# Bennett Dislocation Fracture Combined With a Trapezium Fracture and Advantages of Iselin K-Wire Fixation: A Case Report

**DOI:** 10.7759/cureus.85039

**Published:** 2025-05-29

**Authors:** Amine Hamzaoui, Khalil Ouda, Mouncef Amahtil, Sadougui Mohammed, Abdelkrim Daoudi

**Affiliations:** 1 Orthopedics Department, Centre Hospitalier Universitaire (CHU) Mohammed VI University Hospital, Oujda, MAR; 2 Traumatology-Orthopedics Department, Centre Hospitalier Universitaire (CHU) Mohammed VI University Hospital, Oujda, MAR

**Keywords:** bennett fracture, closed reduction, islen k-wire fixation, thumb mp joint, trapezium fracture

## Abstract

The combination of a trapezium fracture and a Bennett fracture-dislocation is rare in hand trauma, posing both diagnostic and therapeutic challenges. Anatomical reduction and stabilization of the thumb metacarpophalangeal (MCP) joint, along with optimal management, are key to achieving a good outcome. We present the case of a 20-year-old motorcyclist with this lesion, treated with closed reduction using the Iselin K-wire fixation. Clinical and radiological results were satisfactory at six months follow-up. This case highlights the importance of imaging to detect combined lesions and emphasizes the role of minimally invasive fixation techniques for good results.

## Introduction

A Bennett fracture is well-defined as an intra-articular fracture of the first metacarpal, typically consisting of two fragments. Although the palmar oblique ligament may be involved, its injury is not required for this fracture. However, when disrupted, it contributes to the instability of the fracture. The most common mechanism is a fall onto the hand, with the thumb in an abducted or extended position. The association of a Bennett fracture with that of the trapezium is extremely rare [1] and reflects more intense traumatic forces and greater instability. Neglected or untreated trauma can lead to degenerative changes in the CMC joint, compromising long-term function. We present the case of a 20-year-old motorcyclist who presented with a Bennett fracture-dislocation associated with a trapezius fracture. This patient was treated using the Islen K-wire fixation, with a good clinical outcome at the six-week postoperative follow-up.

## Case presentation

**Figure 1:**
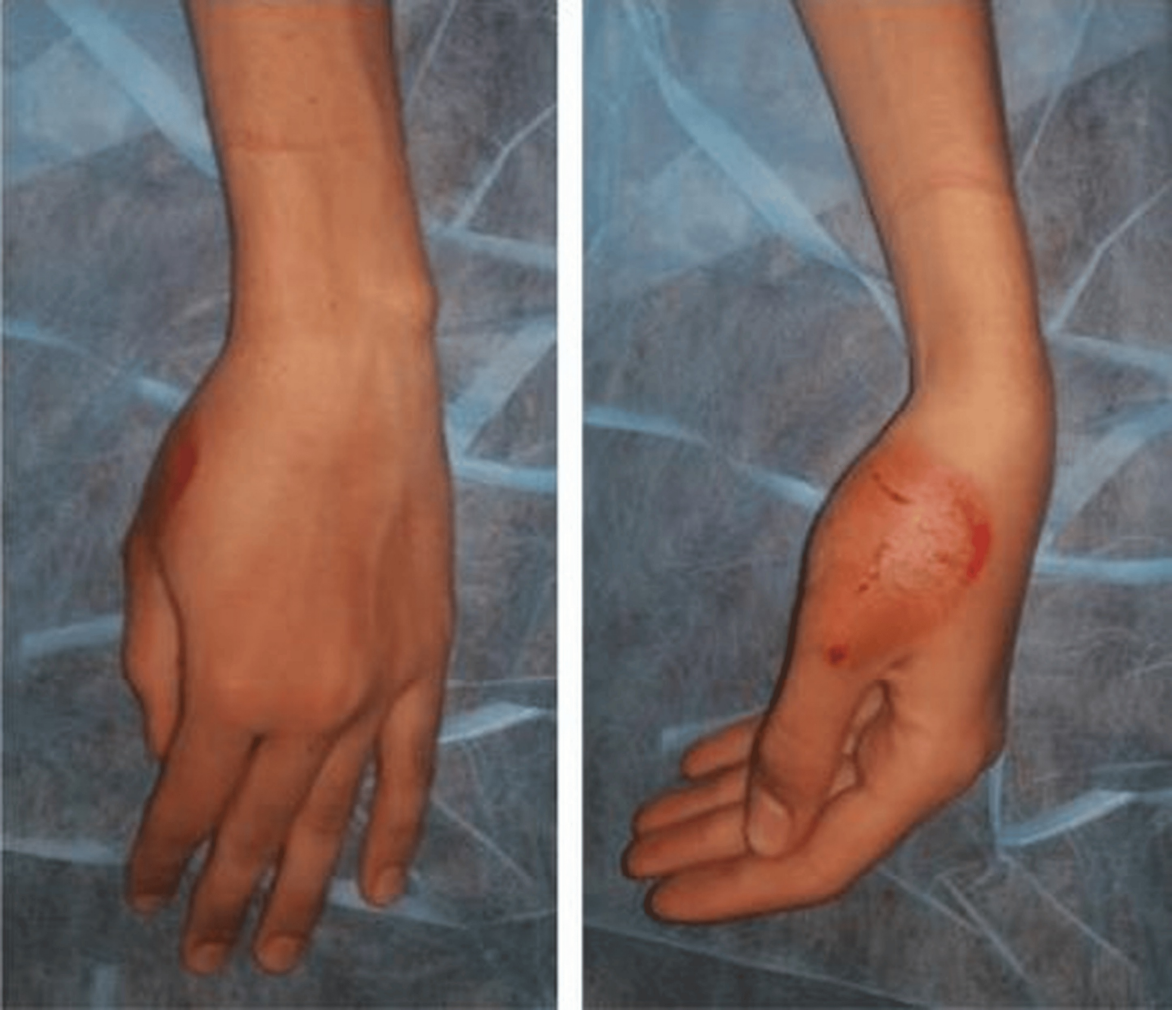
Clinical images of the patient on admission showing deformity and dermabrasion of the base of the thumb.

**Figure 2:**
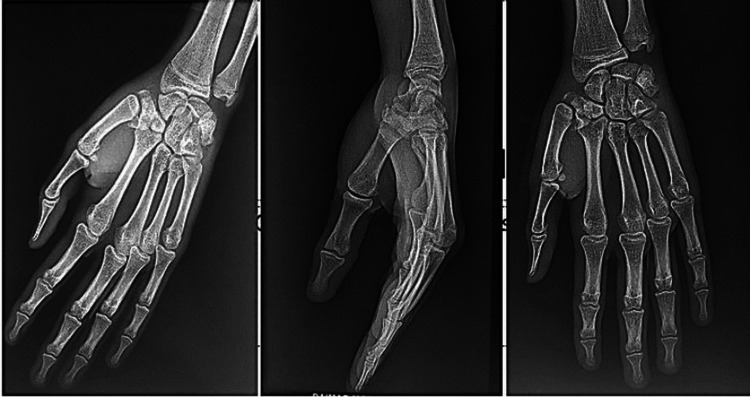
Standard X-ray of the patient showing a trapezium fracture associated with a Bennett fracture-dislocation.

**Figure 3:**
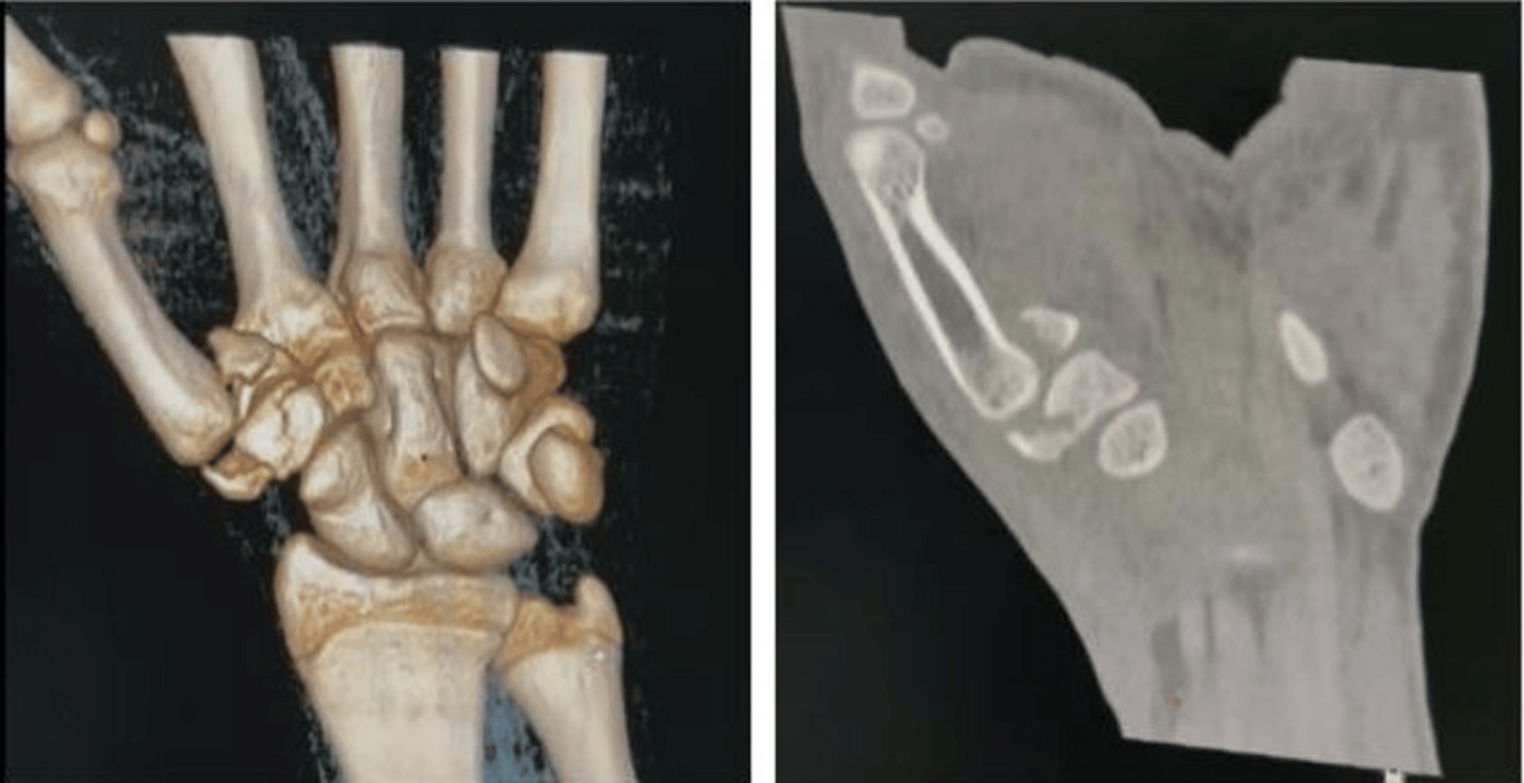
Computed tomography (coronal sections + 3D) of the left hand demonstrating a Bennett fracture associated with a vertical trapezium fracture.

**Figure 4:**
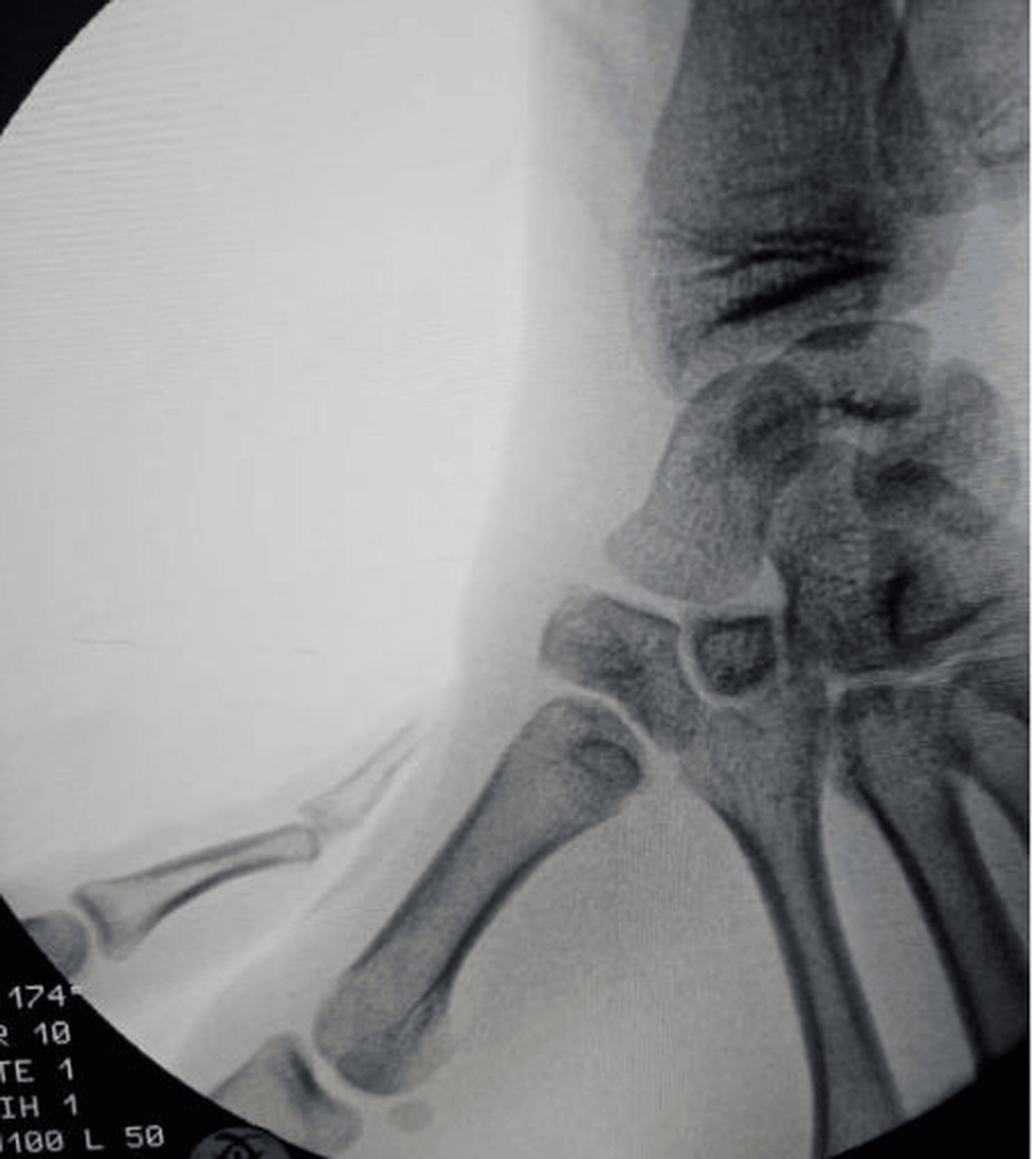
Radiographic fluoroscopy showing reduction through external manipulation.

**Figure 5:**
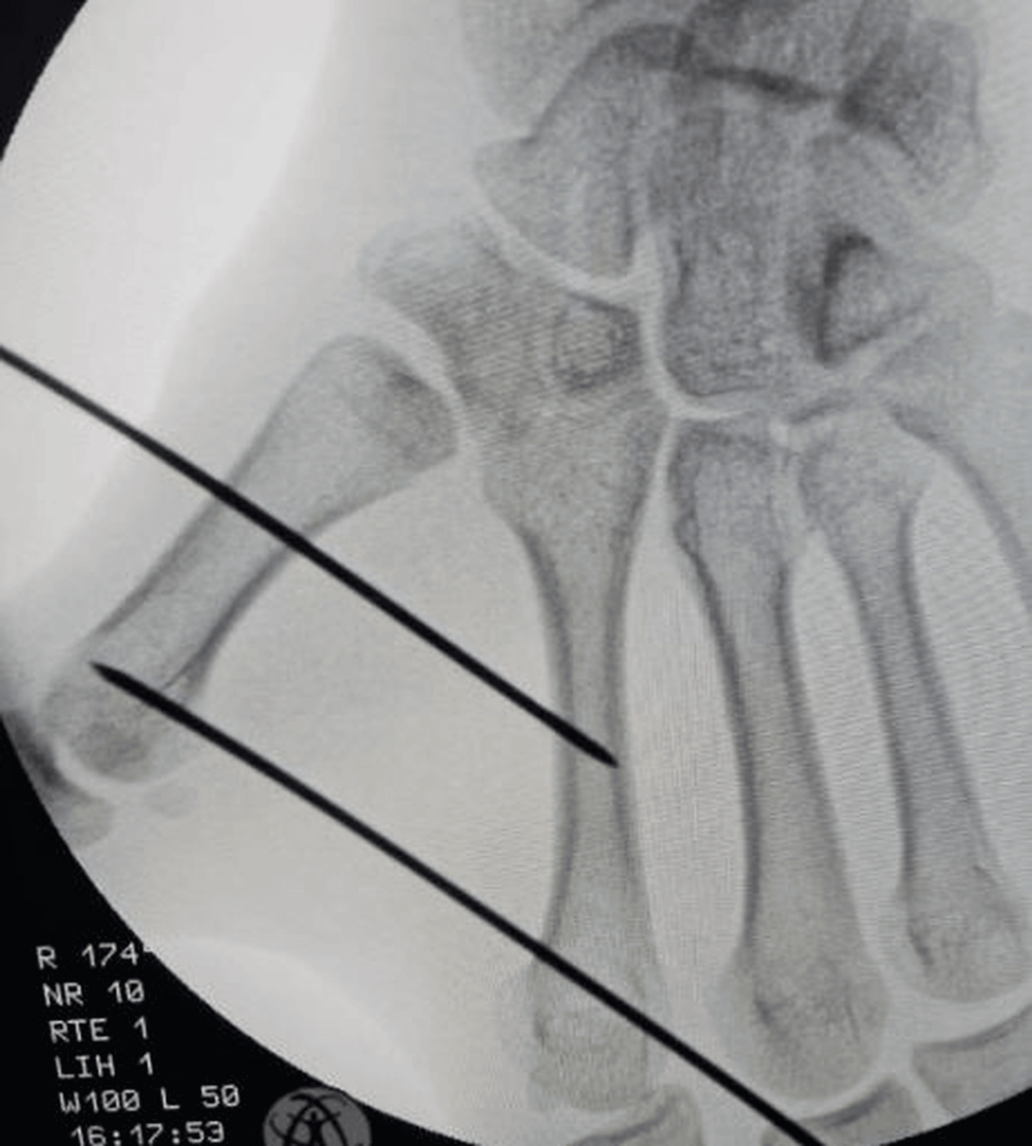
Intermetacarpal K-wire following Iselin’s technique (M2-M1).

**Figure 6:**
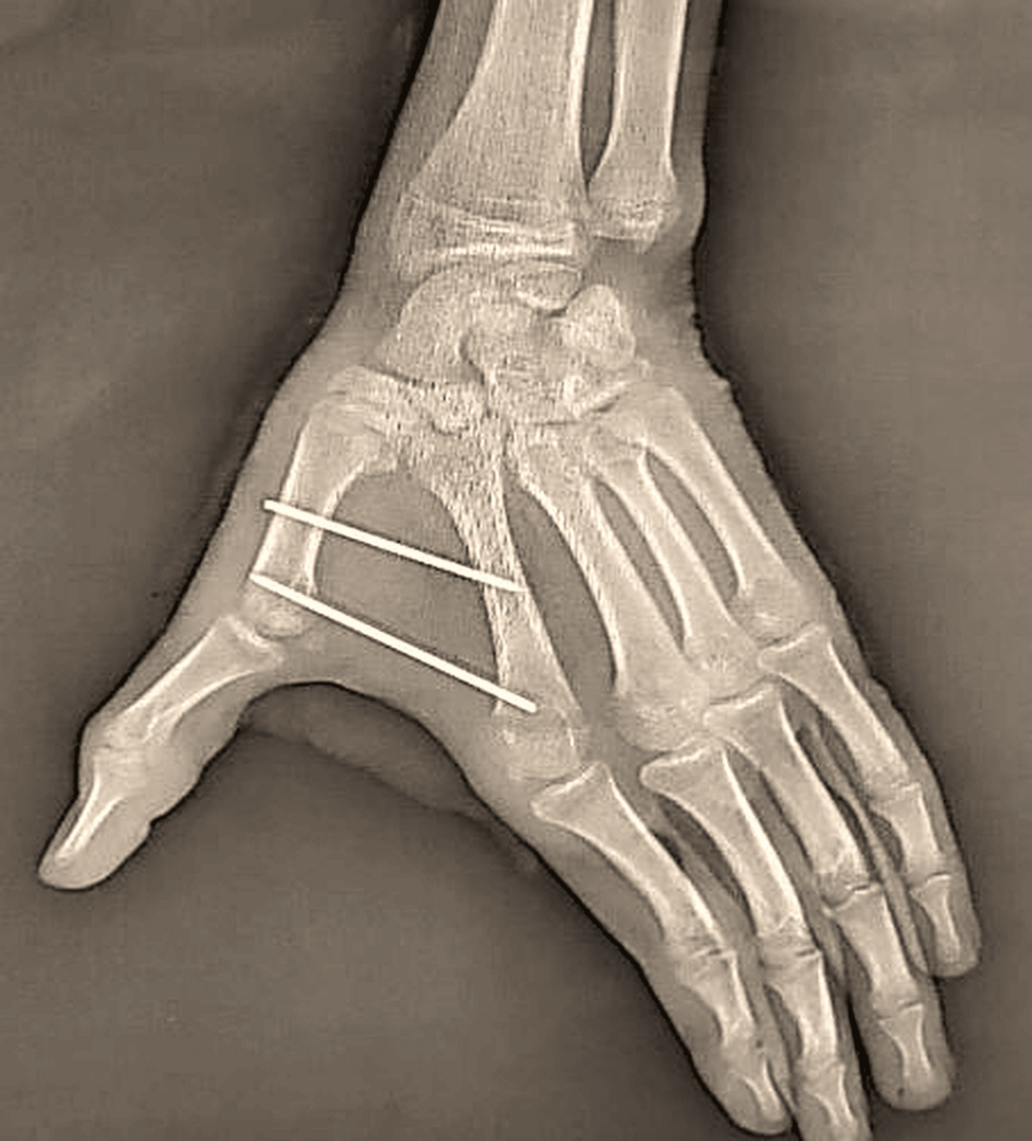
Radiological results at six weeks.

**Figure 7:**
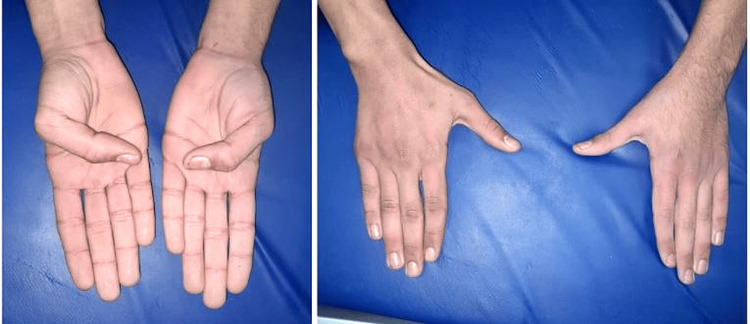
Clinical results and functional outcomes at three months.

We present the case of a 20-year-old right-handed patient who was the victim of a road accident (a motorcyclist hit by a car) with a direct point of impact on the first column of the left hand. Clinical examination revealed edema of the first commissure and thenar eminence, multiple abrasions on the dorsal surface of the lower thumb, and total functional impotence. Neurovascular status was normal (Figure 1). Standard radiographs (Figure 2) revealed a Bennett fracture, characterized by displacement of the medial fragment and dislocation of the carpometacarpal (CMC) joint. This dislocation was associated with a vertical articular fracture of the trapezium, clearly visible on CT scans (Figure 3). The patient was managed surgically under locoregional anesthesia within two hours of the trauma. Closed reductions were performed using external manipulation (Figure 4). The trapezium fracture reduced spontaneously, while stabilization of the Bennett fracture was achieved through percutaneous intermetacarpal K-wire following Iselin’s technique, allowing the opening of the first commissure (Figure 5). Postoperative immobilization with a commissural plaster splint was maintained for six weeks. The fractures healed without complication after six weeks (Figure 6). Rehabilitation was initiated following the removal of the K-wire. The functional outcome was satisfactory after a follow-up of three months based on Kapandji criteria (Table 1), with no pain and good thumb opposition function (Figure 7).

**Table 1:** Kapandji score table

Score	Type of Contact
1	Thumb pulp contacts lateral side of the proximal phalanx (P1) of the index
2	Thumb pulp contacts middle phalanx (P2) of the index
3	Thumb pulp contacts the pulp of the middle finger
4	Thumb pulp contacts the pulp of the ring finger
5	Thumb pulp contacts the pulp of the little finger
6	Thumb pulp contacts the distal interphalangeal (DIP) crease of the little finger
7	Thumb pulp contacts the proximal interphalangeal (PIP) crease of the little finger
8	Thumb pulp contacts the lower palmar crease below the little finger
9	Thumb pulp contacts the lower palmar crease of the hand
10	Thumb pulp contacts the middle of the palm

## Discussion

The trapeziometacarpal joint is a synovial saddle joint with significant mobility, essential for thumb opposition to the other fingers [2,3]. Isolated trapezium fractures are rare or often underdiagnosed, yet they represent the most common fracture of the second row of the carpus [4]. Bennett fractures are associated with approximately 15% of all trapezium fractures [5,6].

Four ligaments stabilize the trapeziometacarpal joint. In the event of dislocation, the dorsoradial ligament ruptures, while the anterior oblique ligament may rupture or remain continuous with subperiosteal tearing. Extending and pronating the thumb relaxes it, facilitating healing [7,8]. But if all the ligaments are ruptured, the joint becomes completely unstable; early open repair is essential, and the function of the dorsoradial and anterior oblique ligaments must be restored [8].

Clinical signs can be subtle and masked by edema, underscoring the need for thorough clinical and radiological evaluation. Standard X-rays are often inadequate for diagnosing trapezium fractures due to radiological overlap with the trapezoid, highlighting the importance of special views described by Kapandji and computed tomography [9]. The goal of treatment is to achieve a mobile, stable, and painless trapeziometacarpal joint to prevent long-term rhizarthrosis. Plaster immobilization with the thumb in an oppositional position provides good outcomes for isolated and non-displaced trapezium fractures [10].

The reduction of these articular fractures can be performed either with open or closed techniques. For articular fractures with large trapezium fragments, osteosynthesis can be achieved using fine Kirschner wires or through screwing with Herbert or Scarf screws [5,6,11]. Despite its technical challenges, osteosynthesis with screws should be preferred. Trapeziectomy may be considered in cases of comminuted fractures [6]. Bennett's fracture, associated with subluxation of the first metacarpal, requires a stabilizing procedure for the thumb column. This osteosynthesis can be performed through closed reduction and percutaneous pinning. Wagner’s pinning technique, which fixes the first metacarpal to the trapezium, cannot be applied in this case. Single intermetacarpal pinning, as described by Johnson, is simple but less robust compared to the double intermetacarpal pinning technique of Iselin [10-12]. Open reduction and internal fixation osteosynthesis allow the restoration of articular surfaces.

## Conclusions

The combination of ipsilateral trapezium and Bennett fractures is uncommon and can go undetected on standard X-rays. Appropriate treatment is required to prevent rhizarthrosis. Percutaneous pinning remains the treatment of choice with good medium-term results.
